# Visual impairment and risk of depression: A longitudinal follow-up study using a national sample cohort

**DOI:** 10.1038/s41598-018-20374-5

**Published:** 2018-02-01

**Authors:** Hyo Geun Choi, Min Joung Lee, Sang-Mok Lee

**Affiliations:** 1 Department of Otorhinolaryngology-Head & Neck Surgery, Hallym University College of Medicine, Anyang, Korea; 2 Hallym Data Science Laboratory, Hallym University College of Medicine, Anyang, Korea; 3 Department of Ophthalmology, Hallym University Sacred Heart Hospital, Anyang, Korea; 4 Department of Cornea, External Disease & Refractive Surgery, HanGil Eye Hospital, 35, Bupyeong-daero, Bupyeong-gu, Incheon, 21388 Korea

## Abstract

The association of visual impairment and depression has been investigated in several studies based on a cross-sectional design, which cannot delineate temporal relationships. In the present study, we evaluated the influence of visual impairment on depression in all age groups using a longitudinal database of a national sample cohort from 2002 to 2013 provided by the Korean National Health Insurance Service. Of a total of 1,025,340 subjects, 5,846 participants who were registered as visually impaired persons without a previous diagnosis of depression were enrolled at a 1:4 ratio with 23,384 control participants matched for age, sex, income, and region of residence. The crude and adjusted (age, sex, income, region of residence, hypertension, diabetes, and dyslipidemia) hazard ratios (HRs) for the development of depression between the visually impaired and control groups were analyzed using a Cox proportional hazards model. Visual impairment increased the risk of depression after adjusting for age, sex, income, region of residence, hypertension, diabetes, and dyslipidemia (adjusted HR = 1.19, *P* = 0.002). The risk of depression increased significantly in both the non-blindness visual impairment (adjusted HR = 1.15, P = 0.036) and blindness subgroups (adjusted HR = 1.31, P = 0.016), with a higher HR in the blindness subgroup.

## Introduction

Functional disabilities, including sensory impairments, are well-known to be associated with depression^1–4^. In particular, the association between visual impairment and depression has been investigated in studies with various designs in different countries using either hospital- or institution-based designs^5–9^, or population-based designs^1–3,10–15^. However, the bidirectional nature of the relationship between visual impairment and depression makes it difficult to delineate a causal relationship because anxiety and depression are also known to affect subjective visual function^15,16^. A thorough objective evaluation of visual function and visual potential minimizes the effect of depression on vision^15^.

Though several studies have investigated the relationship between visual impairment and depression, the majority of previous studies have been based on a cross-sectional design, which does not allow delineation of the temporal relationship between visual impairment and depression^1–4,6–8,10–15^. Therefore, a longitudinal design study is needed to clarify the temporal relationship between visual impairment and depression.

Due to the multifactorial nature of depression, studies on the risk factors for depression are difficult, with numerous confounding factors. Depression is affected by numerous risk factors, including genetics, socioeconomic status/characteristics, social support/relationships, physical health, and functional disability^2,3,17,18^. Therefore, large-scale studies and long-term follow-up data on visually impaired patients, with visual impairment defined by an objective evaluation of visual function/potential, and the comparison of these data with data from a matched control group can help elucidate the temporal relationship between visual impairment and depression.

In Korea, a representative sample cohort database set comprising approximately one million people has been provided by the Korean National Health Insurance Service (NHIS). The database includes medical care histories listed by diagnostic/treatment codes, socioeconomic data, and individual disability and life and death information over a period ranging from 2002 to 2013^19^. The authors concluded that this longitudinal database can be used to clarify the effects of visual impairment on depression because the same database has also been used to describe the effects of hearing impairment on depression^20^.

The purpose of the present study is to evaluate the influence of visual impairment on depression by controlling for possible confounding factors in all age groups using this large, nation-wide, population-based cohort.

## Results

The distributions of age, sex, income level, and region of residence were matched between the visual impaired and control groups (Table 1). The crude hazard ratio (HR) for the development of depression in participants with a visual impairment was 1.22 (95% confidence interval [CI] = 1.09–1.36, *P* < 0.001). After adjustments for age, sex, income, region of residence, hypertension, diabetes, and dyslipidemia, the adjusted HR for the development of depression in these participants was 1.19 (95% CI = 1.06–1.33, *P* = 0.002, Table 2). Following stratification by the severity of visual impairment, the crude and adjusted HRs for the development of depression were significantly higher in both the blindness and non-blindness visual impairment subgroups than in the control group, with an increased HR in the blindness subgroup (non-blindness visual impairment: adjusted HR = 1.15, 95% CI = 1.01–1.31, *P* = 0.036; blindness: adjusted HR = 1.31, 95% CI = 1.05–1.64, *P* = 0.016, Table 2).

**Table 1:** General patient characteristics.

Characteristic	Frequency (n, %)	
	Visual impairment group	Control group
Age group (years)		
0–4	37 (0.6)	148 (0.6)
5–9	41 (0.7)	164 (0.7)
10–14	77 (1.3)	308 (1.3)
15–19	102 (1.7)	408 (1.7)
20–24	201 (3.4)	804 (3.4)
25–29	239 (4.1)	956 (4.1)
30–34	358 (6.1)	1,432 (6.1)
35–39	382 (6.5)	1,528 (6.5)
40–44	111 (1.9)	444 (1.9)
45–49	611 (10.5)	2,444 (10.5)
50–54	667 (11.4)	2,668 (11.4)
55–59	699 (12.0)	2,796 (12.0)
60–64	809 (13.8)	3,236 (13.8)
65–69	647 (11.1)	2,588 (11.1)
70–74	450 (7.7)	1,800 (7.7)
75–79	248 (4.2)	992 (4.2)
80–84	113 (1.9)	452 113 (1.9)
85+	54 (0.9)	216 (0.9)
Sex		
Male	3,481 (59.5)	13,924 (59.5)
Female	2,365 (40.5)	9,460 (40.5)
Income		
1 (lowest)	629 (10.8)	2,516 (10.8)
2	560 (9.6)	2,240 (9.6)
3	433 (7.4)	1,732 (7.4)
4	516 (8.8)	2,064 (8.8)
5	517 (8.8)	2,068 (8.8)
6	467 (8.0)	1,868 (8.0)
7	496 (8.5)	1,984 (8.5)
8	509 (8.7)	2,036 (8.7)
9	545 (9.3)	2,180 (9.3)
10	596 (10.2)	2,384 (10.2)
11 (highest)	578 (9.9)	2,312 (9.9)
Region of residence		
Urban	2,572 (44.0)	10,288 (44.0)
Rural	3,274 (56.0)	13,096 (56.0)
Hypertension		
Yes	2,820 (48.2)	10,379 (44.4)
No	3,026 (51.8)	13,005 (56.6)
Diabetes mellitus		
Yes	1,745 (29.8)	5,136 (22.0)
No	4,101 (70.2)	18,248 (78.0)
Dyslipidemia		
Yes	1,542 (26.2)	6,154 (26.3)
No	4,314 (73.8)	17,230 (73.7)

**Table 2:** Crude and adjusted hazard ratios (95% confidence interval) of visual impairment for depression.

Characteristic	Depression			
	Crude	*P*-value	Adjusted^†^	*P*-value
**Total visual impairment (n = 29**,**230)**				
Visual impairment	1.22 (1.09–1.36)	0.001*	1.19 (1.06–1.33)	0.002*
Control	1.00		1.00	
**Non-blindness visual impairment (n = 23**,**140)**				
Visual impairment	1.17 (1.03–1.33)	0.017*	1.15 (1.01–1.31)	0.036*
Control	1.00		1.00	
**Blindness (n = 6**,**090)**				
Visual impairment	1.37 (1.10–1.70)	0.005*	1.31 (1.05–1.64)	0.016*
Control	1.00		1.00	

*Cox proportional hazard regression model; significant at P < 0.05.

^†^Adjusted model for age, sex, income, region of residence, hypertension, diabetes, and dyslipidemia.

Following stratification by age at enrollment (3 groups: young [0–29 years old], middle-aged [30–59 years old], and elderly [60+ years old]), the crude and adjusted HRs were significantly increased only in the middle-aged population (crude HR = 1.31, 95% CI = 1.11–1.55, *P* = 0.001; adjusted HR = 1.26, 95% CI = 1.07–1.49, *P* = 0.006; Table 3). However, the HR showed a decreasing trend with age (Table 3). Following stratification by sex, the HR for the development of depression was significantly higher in both the male and female subgroups, with similar HRs (male: adjusted HR = 1.19, 95% CI = 1.02–1.40, *P* = 0.029; female: adjusted HR = 1.19, 95% CI = 1.01–1.39, *P* = 0.032).

**Table 3:** Crude and adjusted hazard ratios (95% confidence interval) of visual impairment for depression for age and sex subgroups.

Characteristic	Depression			
	Crude	*P*-value	Adjusted^†^	*P*-value
**Young (0–29 years old**, **n = 3**,**485)**				
Visual impairment	1.39 (0.95–2.04)	0.095	1.30 (0.88–1.92)	0.186
Control	1.00		1.00	
**Middle-aged (30–59 years old**, **n = 14**,**140)**				
Visual impairment	1.31 (1.11–1.55)	0.001*	1.26 (1.07–1.49)	0.006*
Control	1.00		1.00	
**Elderly (60+ years old**, **n = 11**,**605)**				
Visual impairment	1.11 (0.95–1.31)	0.203	1.11 (0.94–1.30)	0.228
Control	1.00		1.00	
**Male (n = 17**,**405)**				
Visual impairment	1.22 (1.04–1.43)	0.012*	1.19 (1.02–1.40)	0.029*
Control	1.00		1.00	
**Female (n = 11**,**825)**				
Visual impairment	1.21 (1.04–1.41)	0.017*	1.19 (1.01–1.39)	0.032*
Control	1.00		1.00	

*Cox proportional hazard regression model; significant at P < 0.05.

Adjusted model for age, sex, income, region of residence, hypertension, diabetes, and dyslipidemia.

## Discussion

In this study, the risk of developing depression among individuals who were registered with a visual impairment was analyzed by comparing these individuals with controls matched for age, sex, income, and region of residence at a 1:4 ratio. The risk of developing depression was significantly increased in the visually impaired population with or without further adjustments for possible confounding factors, including hypertension, diabetes, and dyslipidemia (Table 2, Supplemental Table 1). Additionally, the risk of developing depression was increased significantly in both the blindness and non-blindness visual impairment subgroups, with a higher HR in the blindness subgroup (Table 2).

Although many studies have evaluated the relationship between visual impairment and depression, most used cross-sectional designs that do not allow delineation of the temporal relationship between visual impairment and depression due to the bidirectional relationship between these two factors^1–4,6–8,10–12,14^. Most studies support a significantly higher odds ratio or prevalence of depression in visually impaired patients^3,7,10–12^. However, in some reports, the odds ratio decreased to a statistically insignificant level after controlling for potential confounding factors using multivariate regression models^2,12,15^.

In a study of Chinese subjects aged ≥70 years in Hong Kong, a multivariate stepwise logistic regression analysis identified 16 factors in 4 categories that predicted depression: socioeconomic characteristics, poor social support, functional disability, and poor physical health^2^. Greater levels of depression were observed among individuals with greater financial difficulties, more stressful life events, lower self-perception, less support from friends, and less instrumental support^2^. However, poor vision did not achieve a significant odds ratio (odds ratio = 1.50 relative to subjects with excellent vision, 95% CI = 0.91–2.45). In a cross-sectional study of subjects aged ≥70 years in Britain, the prevalence of depression was significantly higher in visually impaired people than in people with good vision (13.5% *vs*. 4.6%; age- and gender-adjusted odds ratio = 2.69, 95% CI = 2.03–3.56)^12^. However, after controlling for potential confounding factors, including activities of daily living, the odds ratio markedly decreased to a non-significant level (odds ratio = 1.26, 95% CI = 0.94–1.70). In a national survey of US adults aged ≥20 years, the estimated prevalence of depression was higher among visually impaired adults (visual acuity <20/40 in the better eye) than among adults with normal visual acuity (10.7% *vs*. 6.8%)^15^. After controlling for potential confounding factors, the odds ratio of depression in participants with a self-reported loss of visual function remained significant (overall odds ratio = 1.9, 95% CI = 1.6–2.3); however, the odds ratio for visual acuity impairment was no longer statistically significant^15^. As subjective visual function can be affected by depression^16^, this result suggests that the significantly higher odds ratio found in previous studies based on a cross-sectional design and visual function questionnaire cannot be directly interpreted as indicating a temporal/causal relationship between visual impairment and depression. Therefore, a longitudinal design based on an objective evaluation of visual function is strongly needed, which is why this study was designed.

In the present study, we observed a significantly higher HR for the development of depression in the visually impaired population relative to matched controls using a longitudinal study design. This result persisted after controlling for potential confounding factors, including age, sex, income, region of residence and the comorbidities hypertension, diabetes, and dyslipidemia with statistical adjustments using a Cox proportional hazards model.

The subgroup analysis stratified by age revealed that the HR for developing depression was highest in younger subjects (0–29 years old, n = 3,485; adjusted HR = 1.30, 95% CI = 0.88–1.92, *P* = 0.186, Table 3) but was not statistically significant, possibly due to the small size of this population. The HR of the middle-aged population (30–59 years old, n = 14,140) was slightly lower than that of the younger population but was statistically significant (adjusted HR = 1.26, 95% CI = 1.07–1.49, *P* = 0.006, Table 3). In the elderly population (60+ years old, n = 11,605), the HR was lowest and insignificant (adjusted HR = 1.11, 95% CI = 0.94–1.30, *P* = 0.228, Table 3). Overall, the HR of developing depression showed a negative correlation with age, but the subgroup analyses revealed a significantly increased hazard only in the middle-aged population. The decreased HR in the elderly population might be explained by the increased percentage of elderly subjects who developed depression in both the control and visually impaired groups (7.3% [674/9,284] in the control group vs. 8.0% [186/2,321] in the visually impaired group; Supplemental Table 2). The increased percentage of subjects with depression in the control group can be explained by other risk factors that are known to be associated with depression in the elderly population - the presence of other functional disabilities, chronic illnesses including kidney and circulation problems, and socioeconomic factors^2,3,8,13,17,18,21^. However, the percentage of elderly subjects who developed depression in the present study is higher than the prevalence previously reported^7,12,15^. The prevalence of major depressive disorder diagnosed by the fourth edition of The Diagnostic and Statistical Manual of Mental Disorders (DSM-IV) was 5.36% in visually impaired individuals and 1.23% in normal sighted older adults (aged ≥60 years) in the Netherlands^7^. A direct comparison between the percentage of subjects with depression in this study and that among the normally sighted population from previous reports is impossible because the control group in this study is not a real population but a set of control cases for each visually impaired subject matched by age, sex, income, and region of residence at a 1:4 ratio. The percentage in the present study can be interpreted as the cumulative incidence during the study period instead of the prevalence. It is possible that the lower economic status of the control group in the present study, which occurred because of the matching process with the visual impairment group, whose economic status was lower than that of the general population, is another reason for the higher percentage of depression in the control group. This higher percentage can also be explained by the high prevalence of mood disorders in Korea (7.5% lifetime prevalence and 3.6% 12-month prevalence in 2011 as estimated by the DSM-IV criteria)^21^.

Although a higher percentage of female subjects than male subjects developed depression in the subgroup analysis stratified by sex (8.7% [206/2,365] in females compared with 5.8% [201/3,481] in males, Supplemental Table 2), the HRs for developing depression were similar in both sex groups (female: adjusted HR = 1.19, 95% CI = 1.01–1.39, *P* = 0.032; male: adjusted HR = 1.19, 95% CI = 1.02–1.40, *P* = 0.029). This result was observed because the differences in the percentage of subjects with depression between the visually impaired and control groups were similar in both sexes.

One advantage of this study is the design, which allowed the effect of visual impairment on depression to be evaluated in a representative, large-scale sample from a cohort database consisting of one million subjects with a 12-year follow-up period. Subjects who had been previously diagnosed with depression were excluded from both the visually impaired and control populations to elucidate the temporal relationship between these two factors. The analyses benefited from the lack of missing participants in the cohort data, as the dataset is based on claims to the NHIS, the nationwide compulsory health insurance system^19,22^. Additionally, this database represents the entire South Korean population because >97% of South Koreans are covered by the NHIS, and this dataset was created using systematic sampling^23^. Using this large dataset, we were able to choose matched controls, allowing the potential confounding factors of age, sex, income, and region of residence in the development of depression to be controlled for. Additional disease-related confounding factors were also controlled for by retrieving the diagnostic codes of well-known comorbidities.

A second advantage of this study is the precise evaluation of visual function using the government-registered disability data, which are directly related to governmental benefits and involve very strict decision processes regarding visual function/potential. Because depression itself has also been reported to affect subjective visual function^15,16^, we cannot discuss the causal relationship between subjective visual function and depression. The confirmation of visual impairment by objective data is important for the evaluation of the effect of visual impairment on depression.

A third advantage is that the effect of visual impairment was evaluated across all age groups, and stronger effects on depression were observed in young and middle-aged populations. Most previous studies targeted the elderly population^1–5,7,8,10–12,14^, likely because of the higher prevalence and impact of depression in older age groups and because the data are more easily obtained from the pre-existing elderly cohort^7,10–12^. Because young and middle-aged people lead more independent lives, depression can have a greater impact on these patients and the people around them, although the populations are smaller than the elderly population. A previous study that focused on the psychological effects of visual impairment on young and middle-aged individuals reported a beneficial effect of electronic vision aids on psychological well-being, and these aids are more easily adopted by younger individuals^9^. Although there is still only limited evidence for the effectiveness of interventions in visually impaired patients^24^, various psychosocial interventions aimed at decreasing depressive symptoms among visually impaired patients have been recently attempted including problem-solving treatments, active referrals to a physician, and stepped care programs^25–27^. Considering the benefits of newly developed electronic vision aids and the symptomatic improvement of depression by active management despite unimproved visual function, efforts to manage depression should be encouraged for visually impaired patients, including young and middle-aged patients^9,11,25^.

There are some limitations to this study: 1) The diagnostic codes were not available before 2002 for this dataset. 2) The criteria for the diagnosis of depression may vary among psychiatrists. 3) Some conditions that can be related to the development of depression, including stressful life events and emotional support from acquaintances, could not be analyzed together due to the limitations of the existing database. 4) The non-blindness visual impairment subgroup included some participants with monocular blindness according to the criteria for a legal visual disability in Korea. The proportion of monocular blindness included in this study may be concerning. Because grade VI, the mildest grade in the Korean legal visual disability system, includes both Category 0 (monocular blindness in this study, vision in the better seeing eye ≥20/60) and Category 1 (moderate low vision, vision in the better seeing eye 20/70–20/160) based on the World Health Organization (WHO) classification^28^, it is difficult to know the percentage of monocular blindness among the population included in this study. In 2002, 58.2% of the total number of individuals enrolled in the visual disability system were considered grade VI, according to the statistics provided by the Korean government. However, the inclusion of patients with unilateral blindness as their visual impairment does not seem to lower the value of this study because unilateral blindness can also affect the development of depression not functionally but psychologically. 5) Due to the limitations of the case-control design of this study, further evaluations of the confounding factors that affected the results were limited. In a recent longitudinal prospective cohort study of 540 elderly adults with vision impairment, the risk factors for developing depressive symptoms were living alone, reporting reduced health-related quality of life, having macular degeneration, having problems adapting to the vision loss, experiencing symptoms of anxiety, and having just enough money to cover their expenses^18^. Despite all these limitations, we believe that this study reliably shows the temporal relationship between visual impairment and depression, based on its large-scale, longitudinal study design.

In summary, visual impairment increased the risk of depression after adjusting for age, sex, income, region of residence, hypertension, diabetes, and dyslipidemia. The risk of depression increased significantly in both the non-blindness visual impairment and blindness subgroups.

## Methods

### Study Population and Data Collection

The Institutional Review Board (IRB)/Ethics Committee of Hallym University (2014-I148) approved the use of these data. The requirement for written informed consent was waived by the IRB.

This national cohort study relies on data from the Korean Health Insurance Review and Assessment Service - National Sample Cohort (HIRA-NSC)^19,29,30^. The NHIS selects samples directly from the entire population database to avoid non-sampling errors. Approximately 2% of the samples (one million) were selected from the entire Korean population (50 million). The selected data were classified into 1,476 levels (age [18 categories], sex [2 categories], and income level [41 categories]) using randomized stratified systematic sampling methods via proportional allocation to represent the entire population. After data selection, the appropriateness of the sample was verified by a statistician who compared the sample data with the data from the entire Korean population. The detailed methods used to perform these procedures are provided by the National Health Insurance Sharing Service^30^. The cohort database included (i) personal information, (ii) health insurance claim codes (procedures and prescriptions), (iii) diagnostic codes using the International Classification of Disease-10 (ICD-10), (iv) death records from the Korean National Statistical Office (using the Korean Standard Classification of disease), (v) socioeconomic data (residence and income), and (vi) medical examination data for each participant over a period ranging from 2002 to 2013.

Because all Korean citizens are recognized by a 13-digit resident registration number from birth to death, exact population statistics are determined using this database. All Koreans are required to enroll in the NHIS. All Korean hospitals and clinics use the 13-digit resident registration number to register individual patients in the medical insurance system. Therefore, the risk of overlapping medical records is minimal, even if a patient moves from one place to another. Moreover, all medical treatments in Korea are tracked using the HIRA system, without exception. In Korea, a notice of death to an administrative entity is legally required before a funeral can be held.

### Participant Selection

Of 1,025,340 cases with 114,369,638 medical claim codes, we included participants who were registered as visually impaired persons in the Ministry of Health and Welfare. In Korea, legal visual impairment is defined as the presence of any of the following 4 conditions that show stabilization after at least 6 months of treatment and are not reversed by medication or surgery, with the exception of keratoplasty: 1) best-corrected visual acuity (BCVA) ≤20/1000 in the worse eye, 2) BCVA ≤20/100 in the better eye, 3) visual field ≤10° from the visual axis for both eyes, and 4) a binocular visual field <1/2. The patient must submit a medical certificate issued by an ophthalmologist regarding the BCVA, visual field, and the possible reason for visual impairment before being registering as visually impaired. With properly documented evidence of a visual impairment, an assessment committee discusses the feasibility of the visual impairment registration. In Korea, the degree of visual impairment is typically divided into 6 grades according to the severity of impairment; in the database, the data are then divided into two grades (blindness, grades I-II; non-blindness visual impairment, grades III-VI). Blindness is defined as a BCVA ≤20/500 in the better eye, which is compatible with the definition from the WHO^28^.

Those participants with a history of depression prior to visual impairment were excluded from this study (n = 161). Visually impaired participants were matched with participants (control group) who had never been diagnosed with a visual impairment or other disabilities from 2002 through 2013 at a 1:4 ratio. The matches were processed for age, sex, income, and region of residence. Participants in the control group were sorted using a random number order and then selected from top to bottom to prevent selection bias when identifying the matched participants. For example, a participant (60–64 years old, male, income level 1, urban living) could be matched with more than 100 of the control participants who met the same conditions (60–64 years old, male, income level 1, urban living). Then, we selected only 4 control participants from the more than 100 possible control candidates. To do so, we assigned a random number to each of the possible control participants and selected 4 participants after sorting them (from top to bottom) based on these random numbers. Using this procedure, we minimized selection bias and randomly selected 4 control participants. The matched control participants were assumed to have been enrolled in the study at the same time as each matched visually impaired participant. Therefore, participants in the control group who died or were diagnosed with depression prior to matching with the visually impaired participants were excluded. Accordingly, visually impaired participants for whom we were not able to identify a sufficient number of matching participants were excluded (n = 445).

**Figure 1:**
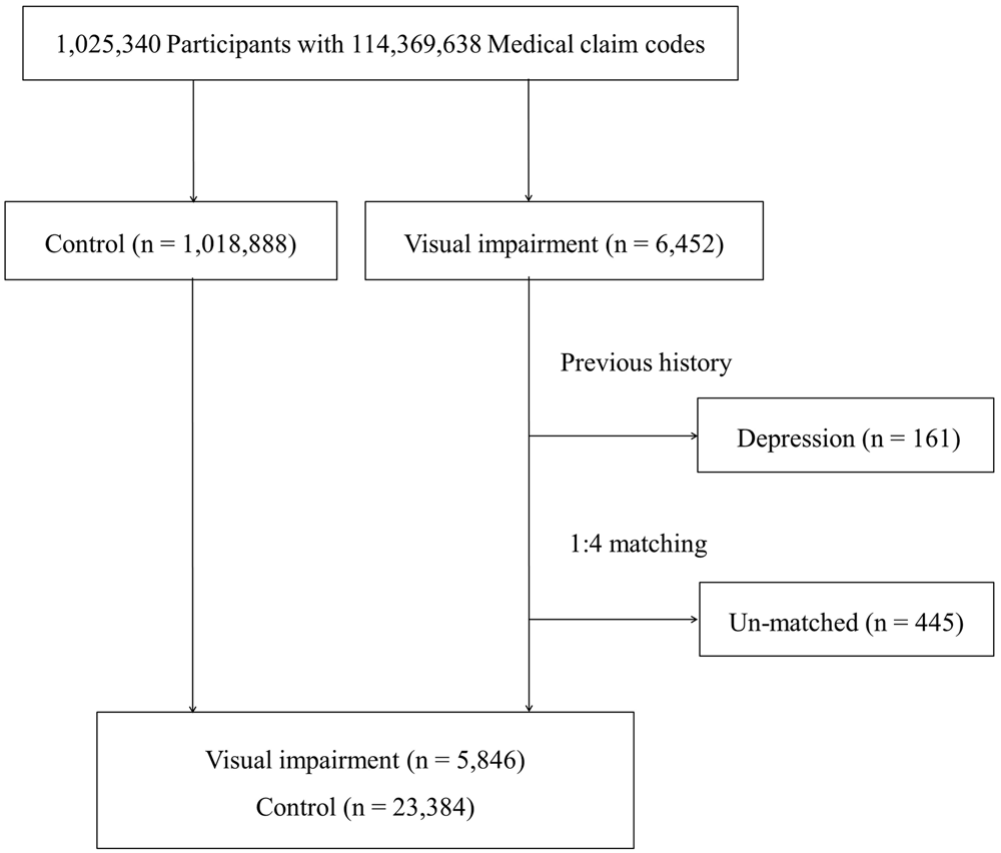
Flowchart of patient selection in the present study. Among a total of 6,452 visually impaired participants, those with a history of depression prior to visual impairment (n = 161) and those who could not be matched with a sufficient number of control participants (n = 445) were excluded. Data from 5,846 visually impaired participants and 23,384 control participants were subsequently analyzed.

Finally, 1:4 matching resulted in the inclusion of 5,846 participants with a visual impairment and 23,384 control participants (Fig. 1). Participants were not matched for past medical history (hypertension, diabetes, and dyslipidemia).

### Variables Used for Matching

The age groups were classified into 5-year intervals: 0–4, 5–9, 10–14… and 85+ years old, resulting in 18 age groups. The income groups were initially divided into 41 classes (one health aid class, 20 self-employment health insurance classes, and 20 employment health insurance classes). These groups were then re-categorized into 11 classes (from class 1 [lowest income] to class 11 [highest income]). The region of residence was divided into 16 areas according to administrative district. These areas were grouped into urban (Seoul, Busan, Daegu, Incheon, Gwangju, Daejeon, and Ulsan) and rural (Gyeonggi, Gangwon, Chungcheongbuk, Chungcheongnam, Jeollabuk, Jeollanam, Gyeongsangbuk, Gyeongsangnam, and Jeju) categories. The past medical histories of the participants were evaluated using ICD-10 codes. For the analysis, participants were listed as having hypertension (I10 and I15), diabetes (E10-E49), and dyslipidemia (E78) if they were treated ≥ 2 times.

Depression was defined by a psychiatrist from 2002 through 2013 using the following ICD-10 codes: F31 (bipolar affective disorder) through F39 (unspecified mood disorder).

### Statistical Analyses

A Cox proportional hazards model was used to analyze the HRs for the effect of visual impairment on depression. In this analysis, crude (simple) and adjusted (age, sex, income, region of residence, hypertension, diabetes, and dyslipidemia) models were used. For the subgroup analysis, we grouped the participants by age or sex (young [0–29 years old], middle-aged [30–59 years old], and elderly [60+ years old]; male and female). Two-tailed analyses were conducted, and *P*-values less than 0.05 were considered to indicate significance. The results were statistically analyzed using SPSS v. 21.0 (IBM, Armonk, NY, USA).

### Data availability Statement

All data is available from the database of Korea national health insurance sharing service (https://nhiss.nhis.or.kr). KNHISS does not allow researchers to provide data to other sites personally. Therefore, the authors do not have the right to provide materials to another person or institution. In order to access the original data of this paper, you can follow the KNHISS guidelines and promise to follow the research ethics through the website, and then provide a certain fee and request the raw data. This process requires IRB approval.

## Electronic supplementary material

Supplemental tables
