# Supplementary material for: Visual impairment and risk of depression: A longitudinal follow-up study using a national sample cohort
Source: Sci Rep. 2018 Feb 1;8:2083. doi: 10.1038/s41598-018-20374-5 (PMC5794974; doi:10.1038/s41598-018-20374-5)
Supplement: Supplementary file 1 — Supplemental tables [file 41598_2018_20374_MOESM1_ESM.pdf]

Visual impairment and risk of depression: A longitudinal follow-up study using a national sample cohort

Hyo Geun Choi, MD,[1,2] Min Joung Lee, MD,[3] Sang-Mok Lee, MD[3]

[1]*Department of Otorhinolaryngology-Head & Neck Surgery, Hallym University College of Medicine, Anyang, Korea*

[2]*Hallym Data Science Laboratory, Hallym University College of Medicine, Anyang, Korea*

[3]*Department of Ophthalmology, Hallym University Sacred Heart Hospital, Hallym University College of Medicine, Anyang, Korea*

Corresponding author and reprints: Dr. Sang-Mok Lee

Department of Ophthalmology, Hallym University Sacred Heart Hospital,

22Gwanpyeong-ro 170beon-gil, Dongan-gu, Anyang-si, Gyeonggi-do, 14068

Republic of Korea

Tel.: +82-31-380-3834

Fax: +82-31-380-3833

Email: lsm10003@gmail.com

Supplemental Table 1 The percentage of depression in the visual impairment and control groups during follow up

Depression	Visual impairment group	Control group	OR (95% CI)	P-value
**Total visual impairment**				
Normal (n, %)	5,349 (92.9%)	22,037 (94.2%)	1.25 (1.11-1.40)	<0.001*
Depression (n, %)	407 (7.1%)	1,347 (5.8%)		
**Non-blindness visual impairment**				
Normal (n, %)	4,328 (93.5)	17,482 (94.4)	1.18 (1.03-1.34)	0.016*
Depression (n, %)	300 (6.5)	1,020 (5.6)		
**Blindness**				
Normal (n, %)	1,111 (91.2)	4,555 (93.5)	1.38 (1.10-1.74)	0.005*
Depression (n, %)	107 (8.8)	317 (6.5)		

* Chi-square test; significant at $P < 0.05$

Supplemental Table 2 The percentage of depression in the visual impairment and control groups stratified by age and sex during follow up

	Visual impairment group	Control group	OR (95% CI)	P-value
**Young (0-29 years old)**				
Normal (n, %)	662 (95.0)	2,687 (96.4)	1.41 (0.95-2.09)	0.088
Depression (n, %)	35 (5.0)	101 (3.6)		
**Middle-aged (30-59 years old)**				
Normal (n, %)	2,642 (93.4)	10,740 (94.9)	1.32 (1.11-1.57)	0.001*
Depression (n, %)	186 (6.6)	572 (5.1)		
**Elderly (60+ years old)**				
Normal (n, %)	2,135 (92.0)	8,610 (92.7)	1.11 (0.94-1.32)	0.215
Depression (n, %)	186 (8.0)	674 (7.3)		
**Male**				
Normal (n, %)	3,280 (94.2)	13,262 (95.3)	1.23 (1.05-1.45)	0.012*
Depression (n, %)	201 (5.8)	661 (4.7)		
**Female**				
Normal (n, %)	2,159 (91.3)	8,774 (92.7)	1.22 (1.04-1.44)	0.016*
Depression (n, %)	206 (8.7)	686 (7.3)		

* Chi-square test; significant at $P < 0.05$
